# Sperm ultrastructure of *Pochazia shantungensis* (Chou & Lu) and *Ricania speculum* (Walker) (Hemiptera, Ricaniidae) with phylogenetic implications

**DOI:** 10.3897/zookeys.880.32810

**Published:** 2019-10-14

**Authors:** Zhen Jiang, Jianing Liu, Daozheng Qin

**Affiliations:** 1 Key Laboratory of Plant Protection Resources and Pest Management of the Ministry of Education, Entomological Museum, Northwest A&F University, Yangling, Shaanxi 712100, China Northwest A&F University Yangling China

**Keywords:** accessory body, mitochondrial derivatives, planthoppers, spermatozoa, taxonomic implications

## Abstract

The sperm ultrastructure of two ricaniid species, *Pochazia shantungensis* (Chou & Lu) and *Ricania speculum* (Walker), was investigated using light and transmission electron microscopy. Both species have monoflagellate sperm, the shape and ultrastructure of the mature spermatozoon of these two species are similar in morphology, and 128 spermatozoa are organized into sperm bundles with their heads embedded in a homogenous matrix forming the spermatodesmata. The individual sperm is filiform and includes the head, neck and flagellum. The head is needle-like, with a bilayer acrosome and an inferior elongated nucleus which is formed of homogeneously compact and electron-dense chromatin. The neck region is indistinct and is comprised of the centriole and centriole adjunct with a homogeneous dense substance. The long flagellum has the typical 9 + 9 + 2 axoneme microtubule pattern and two symmetrical mitochondrial derivatives with an orderly array of cristae flanking both sides, and a pair of well-developed fishhook-shaped accessory bodies. Current evidence shows that ricaniid species have D-shaped mitochondrial derivatives in cross-section and a serrated electron-dense region. The phylogenetic relationship of Fulgoroidea with other superfamilies in Auchenorrhyncha is briefly discussed.

## Introduction

Spermatozoa are highly specialized male gametes in sexually reproductive animals, and are characterized by patterns of rapid and divergent morphological evolution (Birkhead et al. 2009). Comparative morphological and ultrastructural investigations of insect sperm not only contribute to better understanding of the interspecific morphological differences and provide additional characters for taxonomic analysis, but also may help elucidate phylogenies and the evolutionary history of the group (Jamieson 1987, Alves et al. 2006, Dallai et al. 2008, Araújo et al. 2009, 2010, 2011, Vitale et al. 2011, Dallai 2014).

Planthoppers (Fulgoroidea) are among the most dominant and diverse groups of phytophagous hemipterans with 13,600 species worldwide (Urban and Cryan 2007, Bourgoin 2019). Many species in this group are economically significant pests of major agricultural crops due to their high reproductive potential and capacity to transmit plant pathogens (Urban and Cryan 2007). Up to the present, studies on the sperm ultrastructure of planthoppers have addressed four species, including *Nilaparvata lugens* (Stål) and *Muellerianella fairmairei* Perris (in Delphacidae), *Ricania marginalis* (Walker) (in Ricaniidae) and *Cixius nervosus* Linnaeus (in Cixiidae) (Folliot and Maillet 1970, Dai et al. 1996, Tian et al. 2006). It was found that the acrosome complex of *Nilaparvata lugens* was a monolayer and branched-shaped structure and was wrapped by a membrane (Dai et al. 1996). Tian et al. (2006) revealed the axoneme in the sperm tail of *R. marginalis* (Fulgoroidea) consists of 9 + 9 + 2 microtubules. In addition, Soulier-Perkins and Bourgoin (1998) explored the copulatory mechanisms in Fulgoromorpha and found that sexual selection, three modes of deposition and sperm storage occurs within the Fulgoromorpha. Planthoppers transfer sperm directly or by using a spermatophore; transferred into the spermatheca at the bursa copulatrix ductus level within the bursa (Soulier-Perkins and Bourgoin 1998).

Ricaniidae is one of the larger families of the superfamily Fulgoroidea, currently containing 432 species in 64 genera (Bourgoin 2019). Members of this family are distributed widely in the Afrotropical, Australian, Indo-Malayan and Oceania regions, and primarily around the tropics. A few species are major agricultural pests (Bu and Liang 2011). Here we examined the fine morphology and ultrastructure of the sperm of two more ricaniid species, *Pochazia shantungensis* (Chou and Lu) and *Ricania speculum* (Walker) using light and transmission electron microscopy. This study aims to provide additional characters useful for comparison with other species in the family and provides additional foundations for future taxonomic and phylogenetic analyses of Fulgoroidea.

## Materials and methods

### Source of specimens

Adult males of *P. shantungensis* (Chou & Lu) and *R. speculum* (Walker) in Ricaniidae of the superfamily Fulgoroidea (Hemiptera, Fulgoromorpha) (Szwedo 2018) were used in this study. All samples were collected from shrub woodland on the campus of Northwest A&F University, Shaanxi Province, China (34°15.60'N, 108°03.62'E, elev. 562 m) in the peak of the summer in 2016.

### Light microscopy

To determine the total sizes of the spermatozoa of *P. shantungensis* and *R. speculum*, live adult males of these two species were selected. After rapid dissection under a binocular microscope (Motic SMZ-168, China) in a 0.9% physiological saline solution on an ice tray, sperm samples were spread freely before being extracted and mounted in glycerol using clean microscope slides with cover slips. Pictures were taken using a stereomicroscope (LEICA M205 A, Nussloch, Germany). The mean length of sperm and their heads were measured based on five individuals of each species and three sperm from each individual using the Leica Application Suite System Software.

### Transmission electron microscopy (TEM)

Male adults of the two species were dissected in a 2.5% glutaraldehyde solution containing 3% sucrose in phosphate-buffered saline (PBS, 0.1 M, pH 7.2) to obtain the seminal vesicles. The seminal vesicles were then transferred immediately into cold fixative solution at 4 °C overnight. After rinsing with PBS (0.1 M, pH 7.2) for 5, 10, 15, and 20 min, respectively, and 30 min twice thereafter, the samples were post-fixed in 1% osmium tetroxide (in 0.1 M PBS, pH 7.2) at 4 °C for 1.5 h and were then rinsed again with PBS in the same procedure noted above.

Samples were dehydrated in a series of ethanol solutions (30%, 50%, 70%, 80%, and 90% for 15 min and 100% for 20 min twice) and infiltrated overnight in a mixture of LR-White resin (London Resin Company, Reading, U.K.) and alcohol (1:1) followed by infiltration with pure LR-White resin twice (for 4 h and 8 h, respectively) at room temperature. The samples were then incubated at 60 °C for 48 h.

Ultrathin sections (70 nm) were cut with a diamond knife on the Leica EM UC7 ultramicrotome (Leica, Nussloch, Germany), floated with 3% aqueous solution of uranyl acetate for 10–15 min, and refloated with 4% lead citrate solution for 8–10 min. All samples were examined under JEM-1230 transmission electron microscope (JEOL, Tokyo, Japan) or a Hitachi HT7700 transmission electron microscope (Hitachi, Tokyo, Japan) at 80 kV.

## Results

### Pochazia shantungensis

Taxon classificationAnimaliaHemipteraRicaniidae

(Chou & Lu, 1977)

5804BA4F-6A42-5552-B3FD-23F12459D9A2

#### Description.

**Figure 1.**
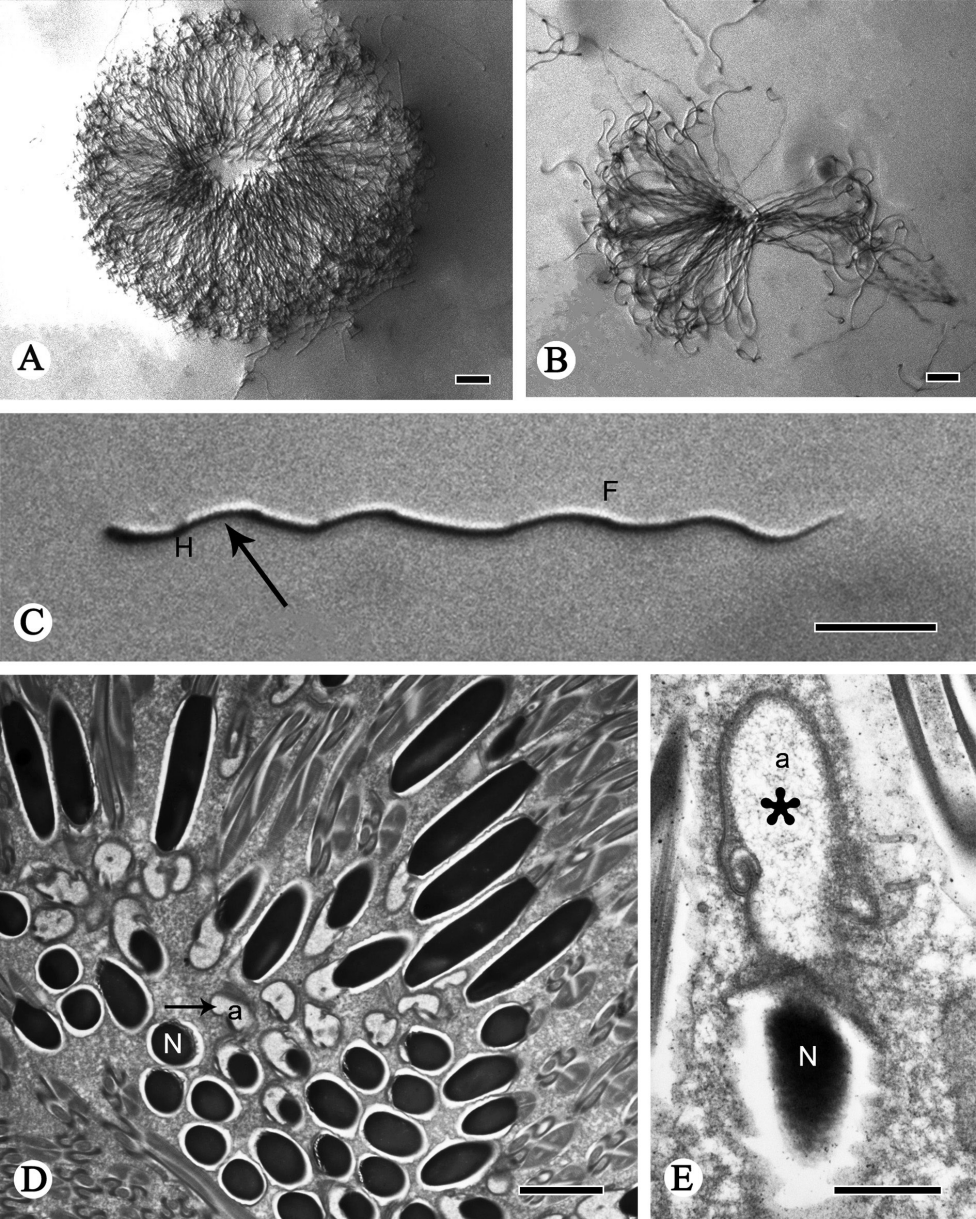
TEM and light micrographs of spermatozoa and spermatodesms of *P. shantungensis*. **A, B** Light micrographs of spermatodesm and spermatozoa **C** light micrograph of a single spermatozoon with the head (**H**, arrow) and wavy ﬂagellum (**F**) **D, E**TEM micrographs of cross-sections of spermatozoa, showing the acrosome (**a**) and nucleus (**N**). Arrow shows head cluster, asterisk indicates the acrosome. Scale bars: 20 µm (**A–C**); 2 µm (**D**); 0.5µm (**E**).

The mature spermatozoa of *P. shantungensis* are held together (totally 128 spermatozoa per spermatodesm) in the form of coiled sperm bundles in the seminal vesicles. Anterior ends of heads are embedded in a homogenous matrix that forms the spermatodesmata (Fig. 1A, B, D). The spermatozoon is long and filiform when it is separated from the bundles and exposed to a 0.9% saline solution (Fig. 1C). It is approximately 127 µm in mean length and has an elongate single head (about 16 µm) and a conventional single flagellum (about 111 µm).

**Figure 2.**
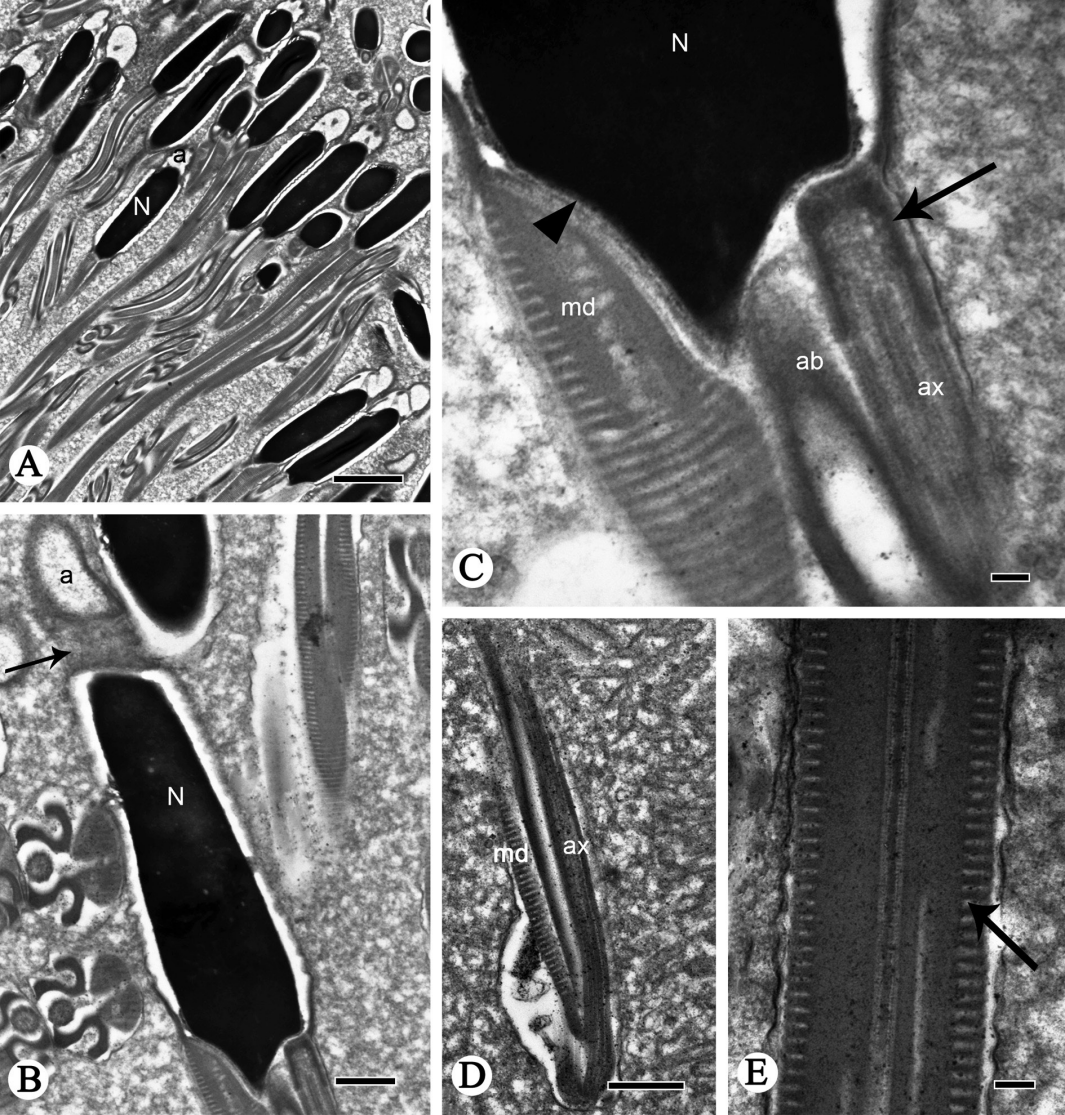
Longitudinal sections of spermatozoa of *P. shantungensis*. **A, B** Spermatozoa, showing acrosome (**a**), nucleus (**N**), arrow indicates connection area between acrosome and nucleus **C** nucleus-flagellum transition, showing nucleus (**N**), mitochondrial derivatives (**md**), accessory body (**ab**), axoneme (**ax**), arrow indicates centriole, triangular arrowhead indicates centriolar adjunct **D, E** flagella of sperm, showing axoneme (**ax**), mitochondrial derivatives (**md**) and cristae (arrow). Scale bars: 2 µm (**A**); 0.5 µm (**B, D**); 0.1 µm (**C, E**).

**Figure 3.**
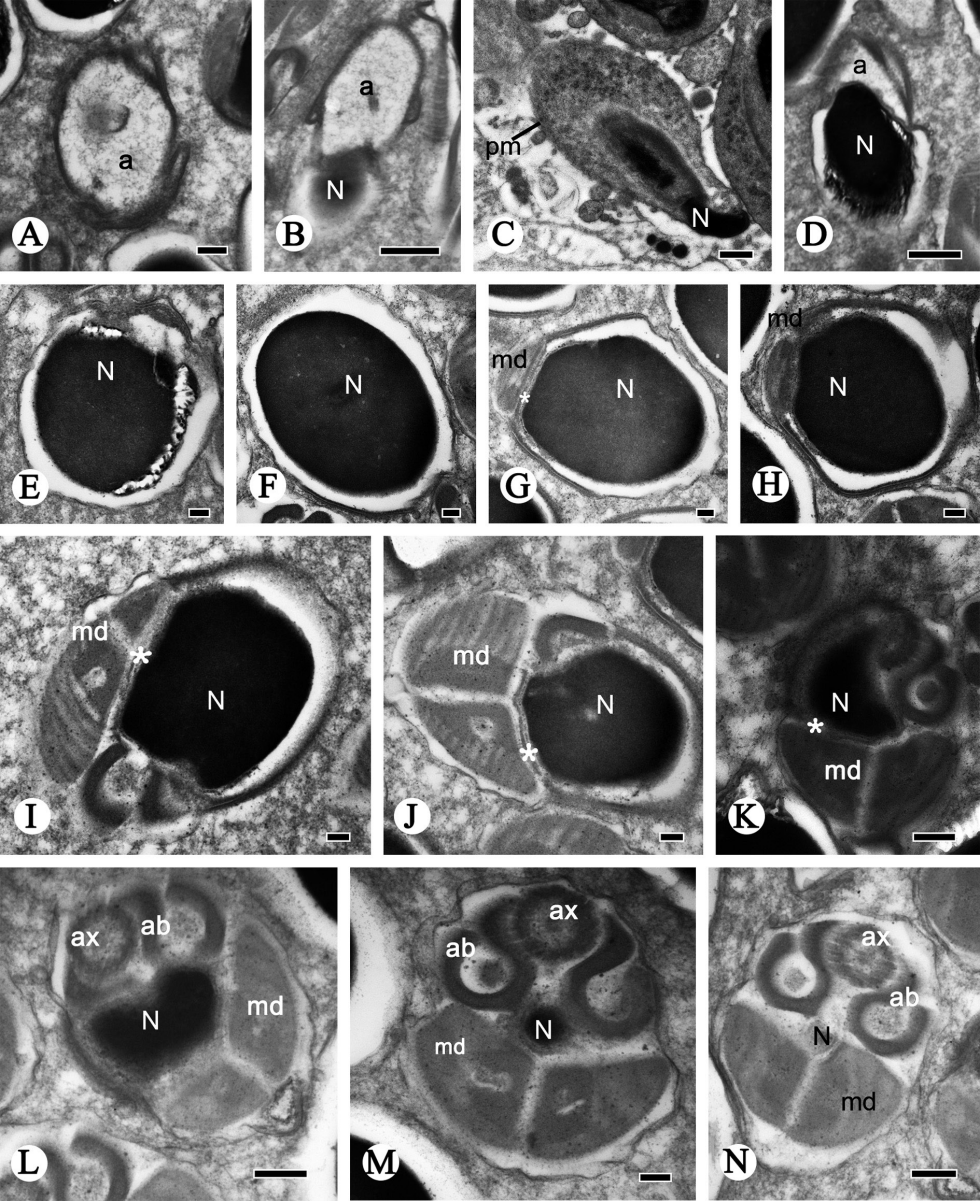
Cross-sections of spermatozoa of *P. shantungensis*. **A** Acrosome, showing the dothideoid acrosome **B–E** serial cross-sections of head showing the dothideoid acrosome (**a**), the nucleus (**N**), and the plasma membrane (**pm**) **F** oval nucleus (**N**) **G–N** nucleus-flagellum transition region, showing the nucleus (**N**), mitochondrial derivatives (**md**), accessory bodies (**ab**), axoneme (**ax**). The asterisk indicates the centriolar adjunct (**ca**). Scale bars: 0.5 µm (**B–D**); 0.2 µm (**A, K, L, N**); 0.1 µm (**E–J, M**).

The head is formed by the nucleus and the acrosome. The acrosome has an irregular saccular acrosomal vesicle and a perforatorium, both located anterior to the nucleus (Figs 1E, 2A, B, 3A, B). Between the base of the perforatorium and the anterior portion of the nucleus is a noticeable transition (Figs 1E, 2B, 3C–E). The acrosome gradually invaginates posteriorly to form a subacrosomal space in which the anterior part of the elongated nucleus is inserted (Figs 2B, 3C, D). The nuclei, different in shape (Figs 2B, 3B–N), are full of homogeneous condensed chromatin and are separated from each other by a cell membrane (Fig. 1D). The ovoid nucleus measures approximately 0.94 µm in diameter in cross-section; in longitudinal-section, it turns into a cylinder-shape (Fig. 2A, B).

The nucleus-flagellum transition region has a centriole and centriolar adjunct (Fig. 2B, C). The centriole starts near the terminal incurvation of the nucleus and terminates anterior of the axoneme (Fig. 2B, C); it is parallel to the moderately electron-dense centriole adjunct (Fig. 2B). The centriole adjunct contains dense granules between the accessory bodies and mitochondrial derivatives in longitudinal profile (Fig. 2B, C); in cross-section the centriole adjunct arises near the end of the nucleus and terminates anterior to the mitochondrial derivatives (Fig. 3I–K). The nucleus is wrapped by the centriolar adjunct (Figs 2B, C, 3I–M). In several transverse profiles of spermatozoa, the posterior nucleus region overlaps the different regions of accessory bodies and mitochondrial derivatives (Fig. 3G–L).

**Figure 4.**
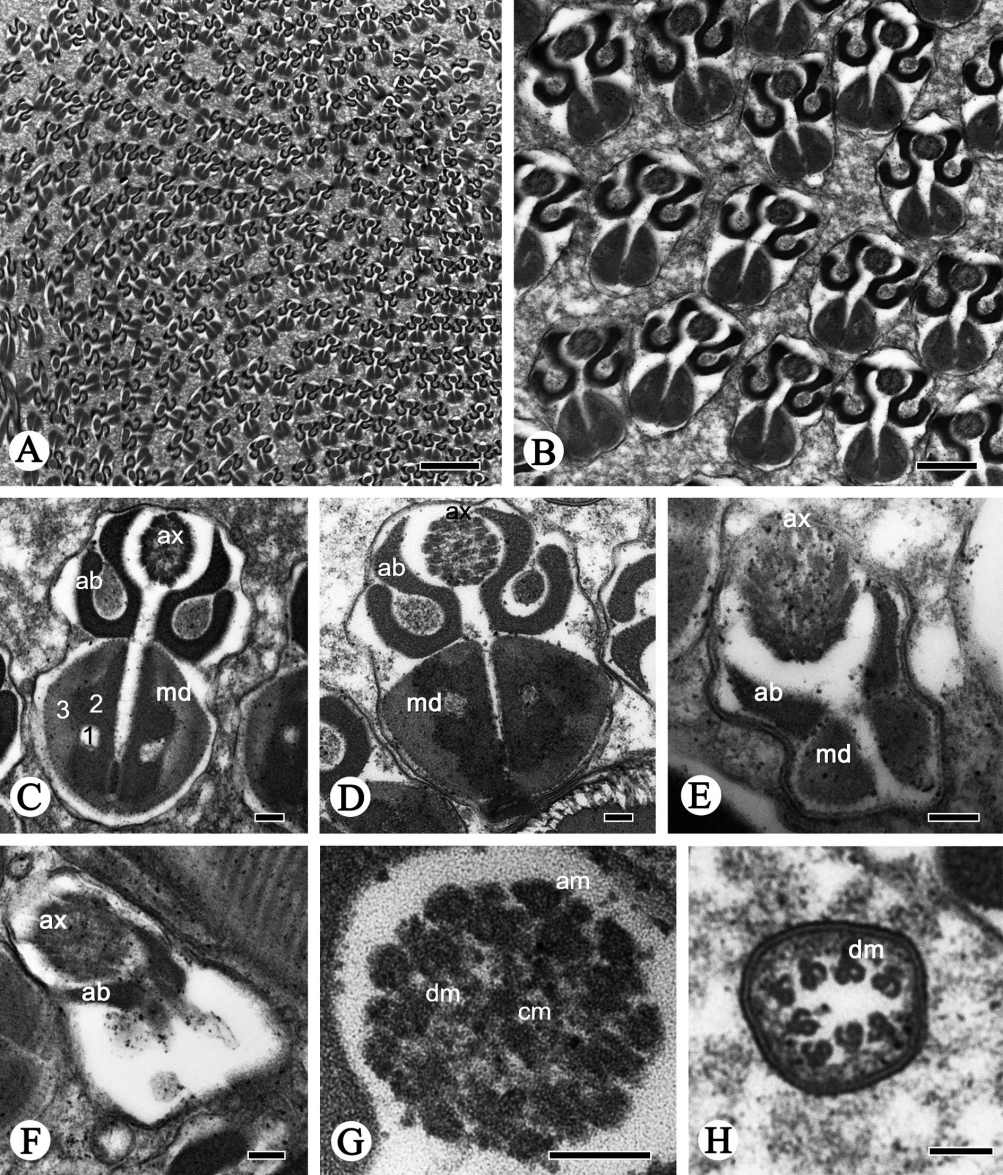
Cross-sections of the sperm flagellum of *P. shantungensis*. **A–D** Flagella, showing axoneme (**ax**), fishhook-shaped accessory bodies (**ab**), D-shaped mitochondrial derivatives (**md**), containing oval lucent region (**1**), serrated electron-dense region (**2**) and mitochondrial cristae region (**3**) **E–F** flagellum, mitochondrial derivatives slowly disappear, axonemes (**ax**) become disordered, accessory bodies (**ab**) become smaller **G** axoneme, showing the typical 9 + 9 + 2 pattern, nine outermost accessory microtubules (**am**), nine doublet microtubules (**dm**) and two innermost central microtubules (**cm**) **H** Showing doublet microtubules finally disappearing. Scale bars: 2 µm (**A**); 0.5 µm (**B**); 0.1 µm (**C–H**).

The flagellum region contains an axoneme, two mitochondrial derivatives and two accessory bodies (Fig. 4A–D); they are parallel to each other throughout most of the length of the flagellum (Fig. 2B–D). The axoneme arises from the centriole (Fig. 2C). It is composed of two innermost microtubules, nine outermost accessory microtubules, and nine doublets, showing the typical 9 + 9 + 2 microtubules arrangement in insects (Fig. 4D, G). The mitochondrial derivatives and accessory bodies are symmetrical in size and diameter in cross-section (Fig. 4A–D). Each mitochondrial derivative is made up of one serrated electron-dense area, one small oval electron-lucid portion and one mitochondrial cristae region (Fig. 4C). In longitudinal-section, the mitochondrial derivatives are positioned lateral to the axoneme and are initiated near the extreme base of the centriole adjunct (Fig. 2B–D). The cristae are perpendicular to the longitudinal axis, bearing regular intervals (42 nm) between adjacent derivatives (Fig. 2E). The accessory bodies are fishhook-shaped; they originate from the centriolar adjunct between the axoneme and mitochondrial derivatives (Fig. 4B–D).

Close to the posterior sperm tip, the axoneme becomes disorganized step by step, and the accessory bodies gradually taper to a cone-shape, while the mitochondrial derivatives disappear (Fig. 4E, F). At the terminal region of the flagellum, the doublet microtubules are the last to disappear (Fig. 4H).

### Ricania speculum

Taxon classificationAnimaliaHemipteraRicaniidae

(Walker, 1851)

82A056D0-D45E-54A4-BD54-516AECC8BEC9

#### Description.

**Figure 5.**
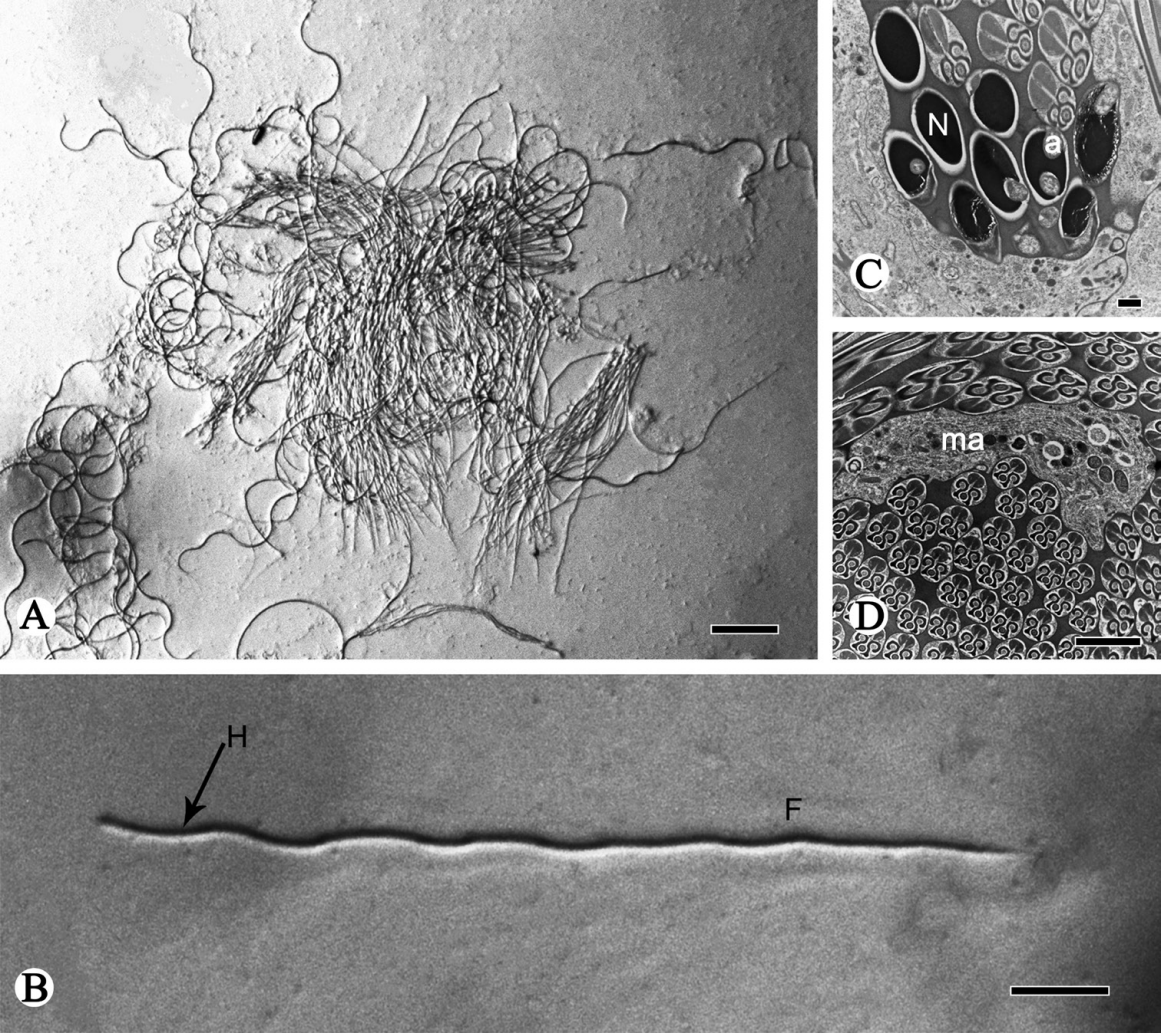
TEM and light micrographs of spermatozoon and spermatodesms of *R. speculum*. **A** Light micrograph of spermatodesm **B** light micrograph of spermatozoon with the head (**H**, arrow) and ﬂagellum (**F**) **C, D** cross-sections of the oval nucleus (**N**), showing the acrosome (**a**) and homogenous matrix (**ma**). Scale bars: 50 µm (**A**); 20 µm (**B**); 0.5 µm (**C**); 2 µm (**D**).

Mature spermatozoa of *R. speculum* are similar to those of *P. shantungensis* in morphology insofar as they also have a number of spermatozoa (totally 128 spermatozoa per spermatodesm) organized into sperm bundles with their heads embedded in a homogenous matrix (Fig. 5A, C–D). The individual sperm is filiform, measuring 196 µm in average length (Fig. 5B), with a linear head and distinct flagellum, approximately 24 µm and 172 µm in length, respectively.

**Figure 6.**
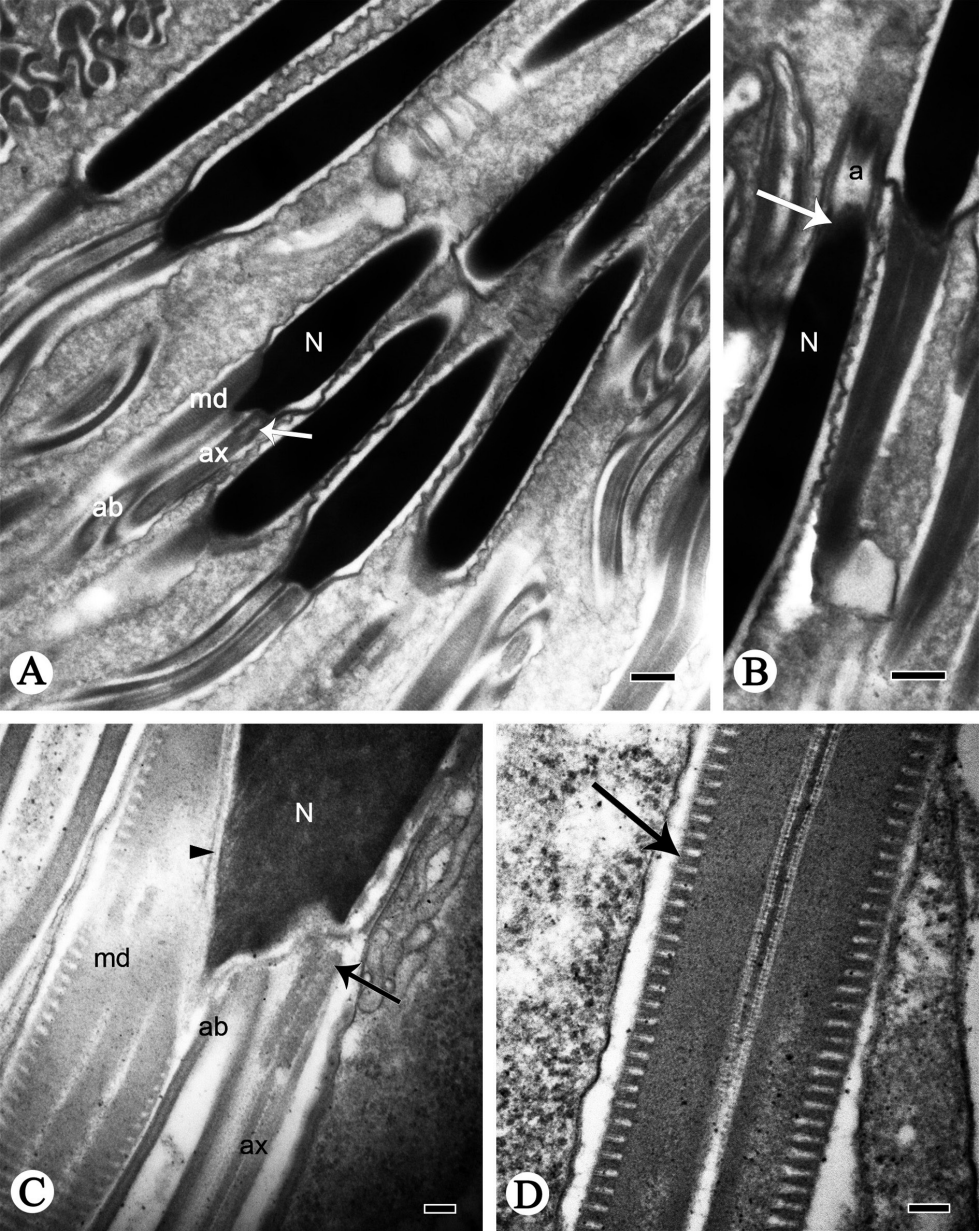
Longitudinal sections of spermatozoa of *R. speculum*. **A, B** Acrosome (**a**), nucleus (**N**), axoneme (**ax**), accessory bodies (**ab**) and mitochondrial derivatives (**md**), arrow indicates acrosome and nucleus connection area **C** nucleus-flagellum transition, showing nucleus (**N**), mitochondrial derivatives (**md**), accessory bodies (**ab**), axoneme (**ax**), arrow indicates centriole, triangular arrowhead indicates centriolar adjunct **D** sperm flagellum, showing cristae (arrow) arranged in mitochondrial derivatives (**md**). Scale bars: 0.5 µm (**A, B**); 0.1 µm (**C, D**).

**Figure 7.**
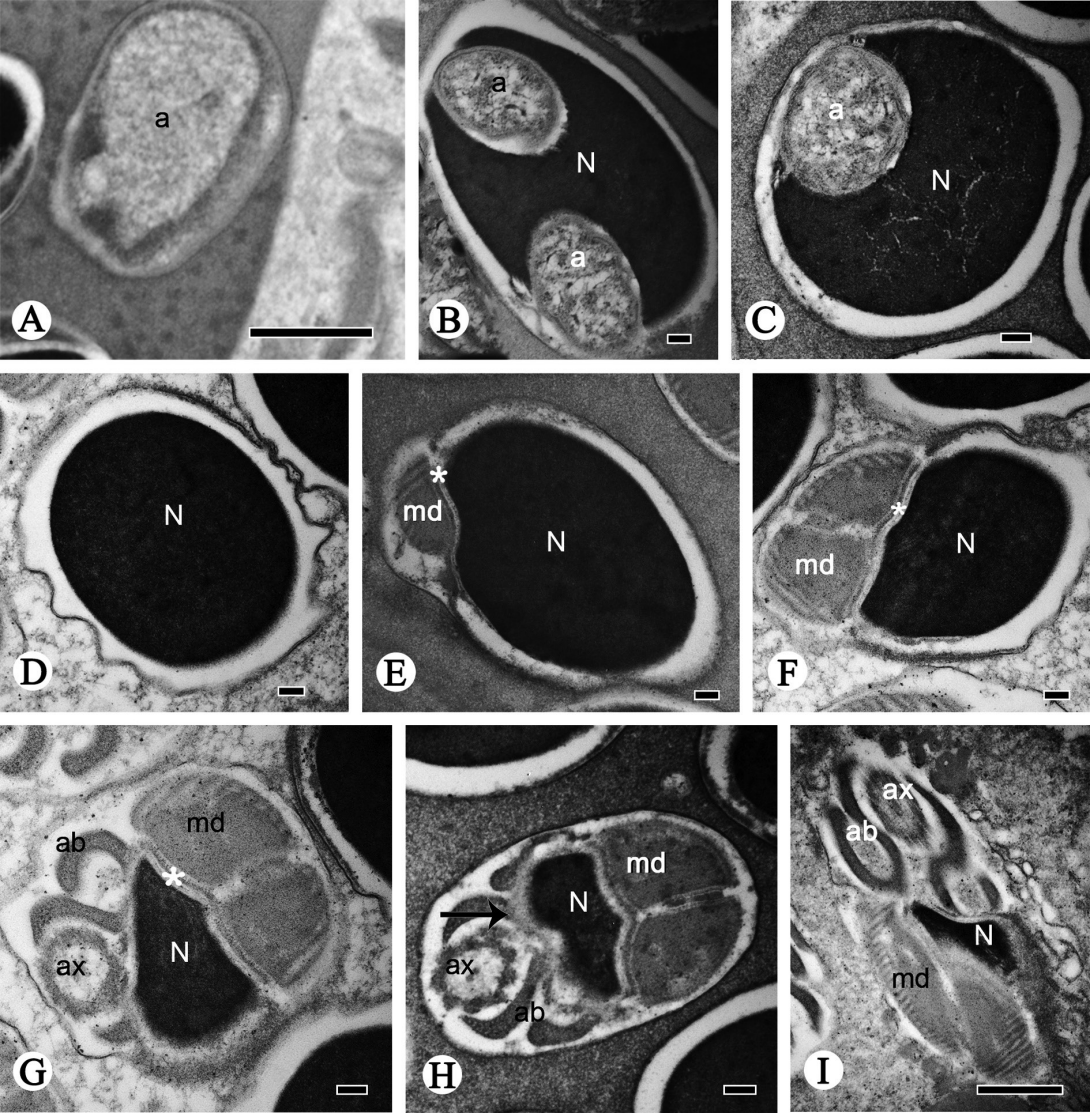
Cross-sections of spermatozoa of *R. speculum*. **A** Showing acrosome (**a**) **B, C** transition region between the acrosome (**a**) and nucleus (**N**), showing acrosome (**a**) on both sides of the nucleus (**N**), until it locates on just the one side of nucleus (**N**) **D** oval nucleus (**N**) **E–I** nucleus-flagellum transition region, showing the nucleus (**N**), mitochondrial derivatives (**md**), accessory bodies (**ab**), axoneme (**ax**), the asterisk indicates the centriolar adjunct (**ca**) and the arrow indicates the centriole (**c**). Scale bars: 0.5 µm (**A, I**); 0.1 µm (**B–H**).

The sperm head of *R. speculum* is elongated and filiform, formed by a short acrosome and an elongated nucleus (Figs 5B, 6B). The conical acrosome contains a dothideoid acrosomal vesicle and perforatorium with the latter made of electron-dense fiber substructures (Fig. 6B). A transition region is visible between the acrosome and the anterior portion of the nucleus (Fig. 6B). Anteriorly the nucleus is surrounded by the acrosome which is filled with numerous fibrous substructures; posteriorly it increases in diameter and changes from a mushroom-shape to a meniscus shape and finally to an oval-shape (Fig. 7B–D). The nucleus, approximately 0.99 µm in diameter, is filled with compact chromatin and takes on different shapes (Figs 6C, 7B–I).

In the nucleus-flagellum transition region, the centriole and centriolar adjunct that lie next to the nucleus are abrupt (Fig. 6A, C). The centriole is formed by dense microtubules that originate from the end of the pyknotic nucleus and end above the front of the axoneme (Figs 6B, 7G–H). The centriolar adjunct is composed of moderate electron-dense substances, connecting mitochondrial derivatives with the nucleus (Figs 6B, 7E–I).

**Figure 8.**
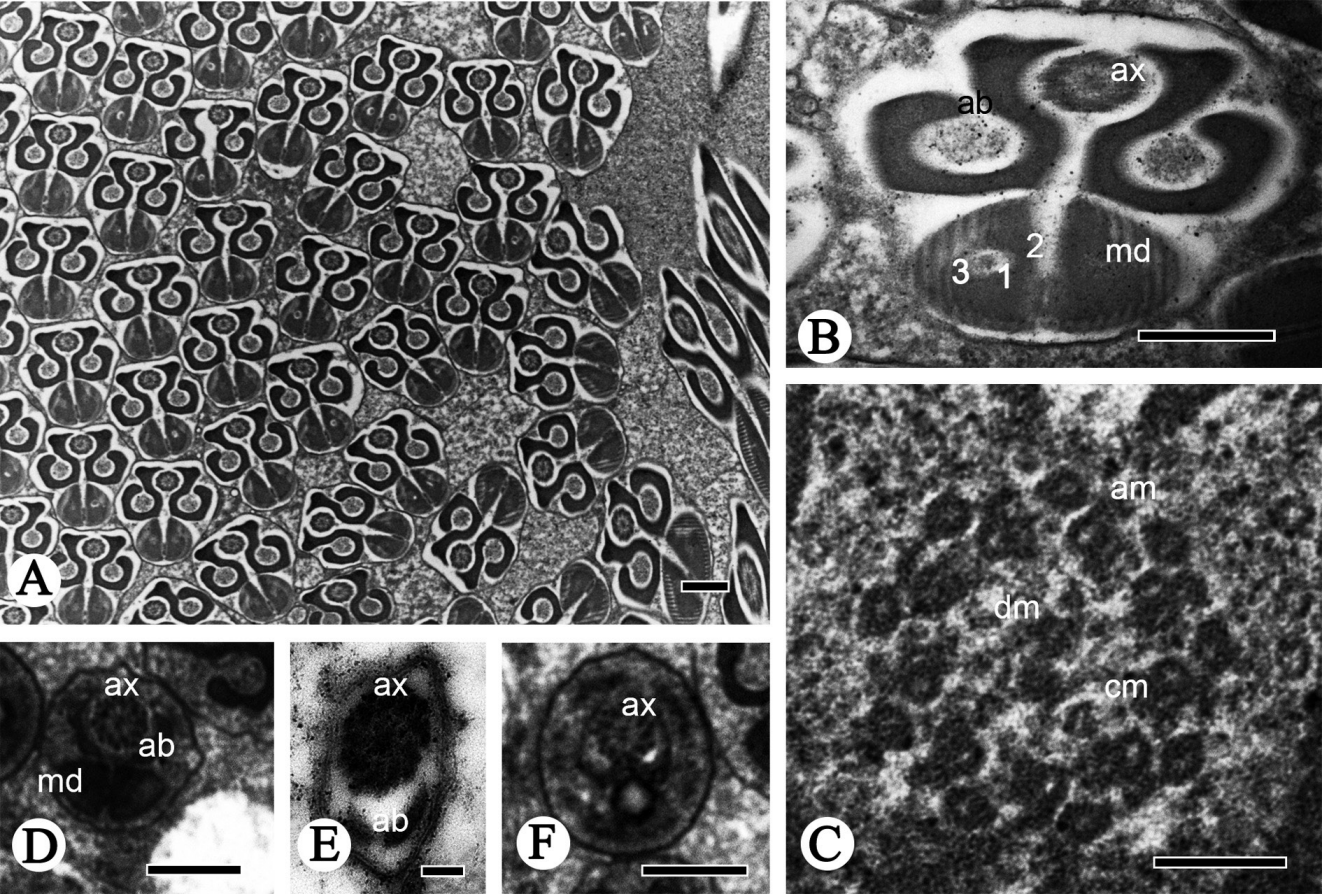
Cross-sections of the sperm flagellum of *R. speculum*. **A, B** Flagella, showing the axoneme (**ax**), accessory bodies (**ab**) and mitochondrial derivatives (**md**) including oval electron-lucid portion (**1**), an electron-dense region (**2**), and one mitochondrial cristae region (**3**) **C** axoneme, showing the typical 9 + 9 + 2 pattern, nine outermost accessory microtubules (**am**), a pair of central microtubules (**cm**), and doublet microtubules (**dm**) in between **D** flagellum, showing the axoneme (**ax**), accessory bodies (**ab**) and mitochondrial derivatives (**md**) **E–F** flagellum without mitochondrial derivatives (**md**). Scale bars: 0.5 µm (**A, B, D, F**); 0.1 µm (**C, E**).

The cross-section of the flagellum region consists of an axoneme, two symmetrical accessory bodies and two mitochondrial derivatives (Fig. 8A, B). The axoneme of the flagellum of *R. speculum* has a typical 9 + 9 + 2 microtubule pattern, comprised of two central microtubules, nine inner doublet microtubules and nine outermost single accessory microtubules (Fig. 8C). The mitochondrial derivatives have evident parallel cristae arranged in the periphery and are formed by three different portions: a serrated electron-dense region, a central clear area and a mitochondrial cristae region (Fig. 8B). The cristae are perpendicular to the axis of the derivatives and are at regular intervals (about 46 nm) between adjacent derivatives (Fig. 6D). Between the axoneme and the mitochondrial derivatives are large, fishhook-shaped accessory bodies (Fig. 8A, B); they are composed of electron-dense material (Fig. 8B). Close to the posterior sperm tip, the mitochondrial derivative is first to end (Fig. 8E), followed by the accessory bodies, while the axoneme gradually becomes disorganized (Fig. 8B, D–F).

## Discussion

This study shows that the mature spermatozoa of these two ricaniid species are similar in quantity and morphology. Both are monoflagellate sperm and both have a straight and needle-like head, an inconspicuous neck, and the conventional long and sinuate flagellum. This study also reveals that *P. shantungensis* and *R. speculum* both have D-shaped mitochondrial derivatives with three particular regions (a serrated electro-dense region, an oval lucent area and mitochondrial cristae region) (Figs 4C, 8B), similar to the previous investigations in other ricaniid species, *Ricania marginalis* (Walker, 1851) and *Euricania clara* Kato, 1932 (Tian et al. 2006, Jiang and Qin 2018). However, there are several differences between *P. shantungensis* and *R. speculum*. For example, the length of the sperm of *P. shantungensis* (about 127 µm) is shorter than in *R. speculum* (about 196 µm). The acrosome of the sperm is cap-shaped in longitudinal-section in *P. shantungensis* (Fig. 2B) but cylindrical in *R. speculum* (Fig. 6B). These differences may provide additional morphological evidence for species recognition in Ricaniidae.

The number, size and cross-sectional shape of mitochondrial derivatives are different in different insect groups (Phillips 1970, Dallai et al. 1995, Lino-Neto and Dolder 2001, Gracielle et al. 2009, Mancini et al. 2009, Gottardo et al. 2012, Dias et al. 2013, Zhang et al. 2016). For most Sternorrhyncha species, individualised sperm is stored in the seminal vesicle. The spermatozoon flagellum is formed by an axoneme, a 9 + 9 + 2 axoneme microtubule, two accessory bodies and two mitochondrial derivatives (absent in Aleyrodoidea and Coccoidea) (Vitale et al. 2011, Labina et al. 2014, Barcellos et al. 2017). However, in Auchenorrhyncha, spermatozoa are aggregated into bundles in the seminal vesicle, intrude into a homogenous matrix to forming a spermatodesm. Current evidence also suggests that sperm structure in Cicadoidea, Cercopoidea, Cicadelloidea and Fulgoroidea have typical characteristics, viz. a cylindrical and bilayer acrosome, a 9 + 9 + 2 axoneme microtubule pattern, two mitochondrial derivatives. Except Cicadoidea and Cercopoidea, other auchenorrhynchans have two accessory bodies (Folliot and Maillet 1970, Cruz-Landim and Kitajima 1972, Kubo-Irie et al. 2003, Chawanji et al. 2005, 2006, Araújo et al. 2010, Zhang and Dai 2012, Su et al. 2014, Zhang et al. 2016, Dallai et al. 2016). It seems that these two suborders (Cicadomorpha and Fulgoromorpha) have undergone different evolutionary routes, which lead to great changes in the axoneme of the flagellum.

Spermatodesmata were described as rope-like in Cicadoidea, Cicadelloidea and Fulgoroidea (Chawanji et al. 2005, Su et al. 2014, Jiang and Qin 2018), and ball-like in Membracoidea and Cercopoidea (Araújo et al. 2010, Zhang and Dai 2012, Hodgson et al. 2016) (Table 1). In Aphididae, Coccoidea and Psylloidea (Sternorrhyncha), spermatodesmata are elongate with the sperm aligned in one direction (Robison 1972, Vitale et al. 2011, Hodgson et al. 2016, Barcellos et al. 2017). It is possible that the rope-like spermatodesm of Cicadoidea, Cicadelloidea and Fulgoroidea could be plesiomorphic. In Coccoidea, spermatozoa transferred into the female tractus via bundles of spermatozoa (Robinson 1977). In Cicadomorpha, spermatozoa are not transferred free but connected to median rods forming spermatodesmes before being deposited into the female bursa copulatrix (Robertson and Gibbs 1937, Maillet 1959, Chevaillier 1962, Boulard 1965). The family Cercopidae exhibit a special spermatodesmata (type I) different from Cicadidae, Ledridae and Ulopidae (type II) (Soulier-Perkins and Bourgoin 1998). Because the anterior ends of the heads in Ricaniidae are embedded in a homogenous matrix that forms the spermatodesmata, it seems that the spermatozoa of Ricaniidae are very likely not free, but delivered in bundles in the female copulatory tractus using a spermatophore containing spermatozoa fixed in a spermatodesmata (type II).

**Table 1.** The main characters of sperm in Auchenorrhyncha.

Taxa	Spermatodesmata	Accessory bodies	Axoneme microtubule	Spermatozoa
Cicadoidea	rope-like	0	9 + 9 + 2	aggregated into bundles, intrude into a homogenous matrix to form a spermatodesm
Cercopoidea	ball-like	0		
Membracoidea	ball-like	2		
Cicadelloidea	rope-like	2		
Fulgoroidea	rope-like	2		

Previous studies have shown that the mitochondrial derivatives of Psylloidea are asymmetric in diameter and filled by paracrystalline material (Gottardo et al. 2016). But they are symmetrical with two different regions, including a paracrystalline region and a less electron-dense region in Fulgoroidea (Cruz-Landim and Kitajima 1972, Chawanji et al. 2005, 2006, Zhang and Dai 2012, Su et al. 2014, Hodgson et al. 2016, Jiang and Qin 2018). This study found a pair of symmetrical accessory bodies in Ricaniidae, like those found in Aethalionidae (in Membracoidea) and Cicadellidae (Kubo-Irie et al. 2003, Araújo et al. 2010, Zhang and Dai 2012, Su et al. 2014, Zhang et al. 2016). In Aethalionidae, it has also three different regions (a clear, less electron-dense region, a dense area and a mitochondrial crista region) (Araújo et al. 2010). Because recent phylogenetic analyses support the major relationships as Fulgoroidea + (Membracoidea sister to Cicadoidea + Cercopoidea) (Cryan 2005, Johnson et al. 2018), if in Membracoidea the mitochondrial derivatives are 3-parted as in Ricaniidae, it is very probable that Cicadoidea, Cercopoidea and Cicadelloidea have the the apomorphic state, and Ricaniidae share a plesiomorphic state with Aethalionidae. Recent studies also show that ricaniid species have the D-shaped mitochondrial derivatives in cross-section and a serrated electron-dense region, the accessory body large and fishhook-shaped in cross-section, which is different from Aethalionidae and Cicadellidae (Kubo-Irie et al. 2003, Araújo et al. 2010, Zhang and Dai 2012, Su et al. 2014, Jiang and Qin 2018). We think it is probably a peculiar character and could be considered as synapomorphic of the species in this family. However, more spermatological evidence (especially the characters of mitochondrial derivatives and accessory bodies) would still be needed for phylogenetic analysis of Ricaniidae.

## Supplementary Material

XML Treatment for Pochazia shantungensis

XML Treatment for Ricania speculum
